# Initial Events in Bacterial Transcription Initiation

**DOI:** 10.3390/biom5021035

**Published:** 2015-05-27

**Authors:** Emily F. Ruff, M. Thomas Record, Irina Artsimovitch

**Affiliations:** 1 Department of Biochemistry, University of Wisconsin-Madison, 1101 University Ave. Madison, Madison, WI 53706, USA; E-Mail: eruff@wisc.edu; 2 Departments of Chemistry and Biochemistry, University of Wisconsin-Madison, 1101 University Ave. Madison, Madison, WI 53706, USA; 3 Department of Microbiology, The Ohio State University, 105 Biological Sciences, 484 W 12th Ave, Columbus, OH 43210, USA; 4 Center for RNA Biology, The Ohio State University, 105 Biological Sciences, 484 W 12th Ave, Columbus, OH 43210, USA

**Keywords:** RNA polymerase, promoter, kinetics, mechanism, transcription regulation

## Abstract

Transcription initiation is a highly regulated step of gene expression. Here, we discuss the series of large conformational changes set in motion by initial specific binding of bacterial RNA polymerase (RNAP) to promoter DNA and their relevance for regulation. Bending and wrapping of the upstream duplex facilitates bending of the downstream duplex into the active site cleft, nucleating opening of 13 bp in the cleft. The rate-determining opening step, driven by binding free energy, forms an unstable open complex, probably with the template strand in the active site. At some promoters, this initial open complex is greatly stabilized by rearrangements of the discriminator region between the −10 element and +1 base of the nontemplate strand and of mobile in-cleft and downstream elements of RNAP. The rate of open complex formation is regulated by effects on the rapidly-reversible steps preceding DNA opening, while open complex lifetime is regulated by effects on the stabilization of the initial open complex. Intrinsic DNA opening-closing appears less regulated. This noncovalent mechanism and its regulation exhibit many analogies to mechanisms of enzyme catalysis.

## 1. Introduction

**Figure 1:**
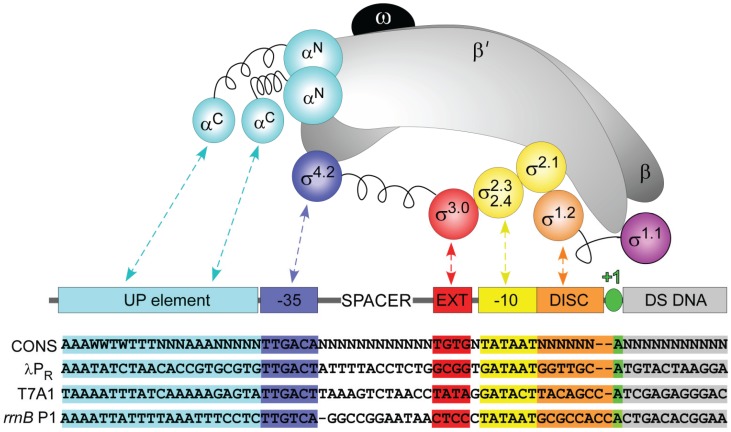
Sequence-specific interactions between σ^70^ RNAP and regions of the promoter. Schematic representations of the subunits of RNAP core, σ^70^, and promoter DNA. RNAP: α_2_: cyan; β and β': gray; ω: black. σ regions: as shown. Promoter: UP element: cyan; −35 element: blue; extended −10: red; −10 element: yellow; discriminator: orange; transcription start site: green; DNA downstream of the transcription start site: gray. Linker regions in α and σ subunits are shown as springs. Nontemplate strand sequences of a “consensus” and λP_R_, T7A1 and *rrn*B P1 promoters are shown below; missing bases are indicated by dashes.

**Figure 2:**
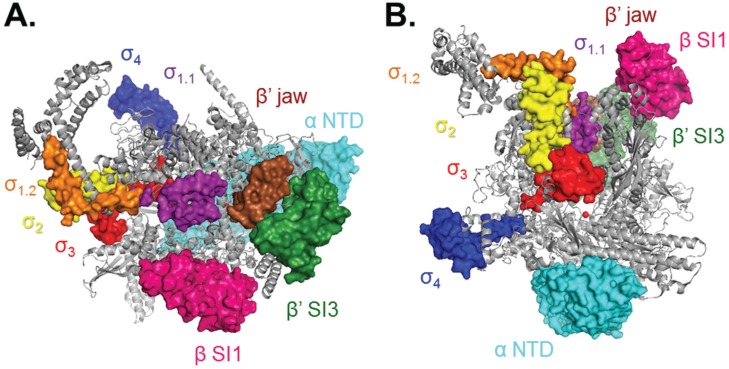
Structural representation of key functional regions of Eσ^70^ holoenzyme. Structure created from PDB 4LK1, looking down the cleft in (**A**) and rotated 90° into the page and 90° counterclockwise in (**B**). Colors of regions of σ are the same as in Figure 1. Additionally shown: β' jaw (β' 1149–1990), brown; β' SI3 (β' insert 6; β' 943–1130), green; β SI1 (β insert 4; β 225–343), pink; active site Mg^2+^, red ball; αNTD (α 1–234), cyan.

Transcription is the first step of gene expression and is therefore one of the most fundamental processes of life. All living organisms, as well as many viruses, encode at least one RNA polymerase (RNAP) enzyme that synthesizes an RNA copy of the template DNA; the core structure and many mechanistic and regulatory elements are shared by bacterial, eukaryotic and archaeal RNAPs (reviewed in [1,2]). Bacterial RNAPs are composed of a core enzyme, which carries out RNA synthesis, and a specificity (σ) subunit for recognition of promoter DNA sequence and subsequent events of initiation (Figure 1 and Figure 2). Specific binding to promoter DNA forms an initial closed complex and sets in motion a series of large conformational changes which bend the downstream duplex DNA into the active site cleft of RNAP and then open the DNA to form a transcription bubble, placing the template DNA strand into the active site. At some (but not all) promoters, subsequent conformational changes in RNAP and the nontemplate strand stabilize this initial open complex (reviewed in [3]). Many steps of this process are highly regulated by promoter DNA sequence, accessory protein factors and small ligands, nucleotide concentration, temperature, salt and solute concentrations, and other environmental variables; aspects of regulation are reviewed in [1,4,5,6,7].

A detailed understanding of the mechanism of initiation and its regulation is centrally important throughout biology, with many applications in biotechnology, pharmacology, and medicine. Determination of noncovalent mechanisms like that of open complex formation and stabilization require both kinetic and structural studies. High resolution structural studies provide detailed information about reactants and products, as well as any intermediates that can be trapped and stabilized [8,9,10,11,12,13,14,15,16,17], but intermediates in open complex formation have been challenging to obtain and characterize in this way. Kinetic studies are used to investigate effects of promoter and RNAP variants, regulators, and regulatory variables [18,19,20,21,22], as well as to establish reaction conditions necessary to obtain transient high concentrations (bursts) of key intermediates for characterization by footprinting and other methods [23,24]. Real-time DNA footprinting provides snapshots of the population of intermediates (and any reactants and products) at any given time, as well as kinetic information about the change in the distribution of these species with time [23,25,26,27]. Ensemble and single-molecule experiments with fluorescent probes can provide unparalleled combinations of structural, dynamic and kinetic-mechanistic information [21,28,29,30,31,32,33,34,35].

Studies with *Escherichia coli* RNAP reveal that the early steps of open complex formation, including initial specific binding to the promoter and some or all of the coupled conformational changes that bend DNA into the cleft, are often rapidly reversible in comparison to the slower “isomerization” step that includes DNA opening and is the rate-determining step of open complex formation. The forward direction of the subsequent large conformational changes that stabilize the initial open complex are faster than the “bottleneck” opening step, and hence must be investigated by dissociation kinetic and mechanistic studies starting with the stable open complex. In the dissociation direction, these conformational changes are reversible on the time scale of the rate-determining DNA closing step. These and other aspects of this noncovalent RNAP-promoter mechanism make it formally analogous to mechanisms of enzyme (covalent) catalysis, wherein binding of substrate (or product, in the reverse direction) and subsequent conformational changes are typically rapidly reversible on the time scale of the rate-determining covalent catalytic step that, like noncovalent DNA opening, occurs in a local environment in the active site. For enzyme-catalyzed reactions, most regulation by inhibitors, activators, and allosteric effectors, as well as the cooperativity of multisite enzymes, occurs in the reversible initial binding steps, while the central catalytic step is relatively insensitive.

In this review, we first discuss the status of the mechanism of forming and stabilizing the open complex between the *E. coli* σ^70^ RNAP holoenzyme and promoter DNA, including what is known about the key RNAP structural and DNA sequence determinants of the rates and equilibria of the steps of this mechanism. We then briefly discuss implications of this mechanism for regulating the rate of open complex formation.

## 2. Bacterial RNAP σ^70^ Holoenzyme

*E. coli* RNAP core enzyme is a five-subunit 370 kDa assembly (α_2_β'βω) [36]; for an extensive review of this structure, see [37]. RNAP is shaped roughly like a crab claw, with an active site cleft running between the β' and β subunits [38]. Although the active site and the overall crab claw shape of RNAPs are highly conserved across all kingdoms [39,40], much of the enzyme is not. The surface of the RNAP is highly divergent, and in many organisms large insertions are present in the β, β' and σ subunits [41]. The functions of many of these regions have yet to be identified. 

RNAP core enzyme carries out all steps of transcription except promoter-specific initiation, which requires an accessory σ factor. There are seven σ factors in *E. coli*, which mediate transcription of distinct sets of genes (reviewed in [5,42]). Holoenzyme containing σ^70^ (termed the “housekeeping σ”) transcribes most of the genes required for growth and cell maintenance, whereas others have more specialized roles, e.g., during stress and nutrient limitation. By itself σ^70^ is incapable of promoter binding and DNA opening. Binding to RNAP core enzyme unmasks specificity determinants in σ^70^ [14,16] and creates the functional holoenzyme.

As part of holoenzyme, σ^70^ recognizes a promoter composed of two well-conserved sequence elements, the −10 and −35 hexamers (Figure 1), which are separated by a 16–19 bp spacer region. σ^70^ is folded into four domains connected by flexible linkers [43]. Upon binding of σ to core, the domains remain independently folded while the linkers extend [13,14,16]. Each domain of σ^70^ interacts with both RNAP and DNA; domains 2 and 4 make specific contacts with the −10 and −35 regions, respectively [43], while domain 3 interacts with the extended −10 region present in some promoters [44].

The flexible N-terminal σ^70^ region 1.1 (σ_1.1_; Figure 2, purple) consists of a three-helix bundle connected by a flexible linker to σ_1.2_ [45]. σ_1.1_, found only in the homologous group 1 σ factors, autoinhibits free σ factor from binding to DNA in the absence of core [46] and occupies the active site cleft in the absence of DNA [8,12]. Kinetics experiments with RNAP lacking σ_1.1_ indicate that this mobile element increases both the isomerization rate (including DNA opening) [47] and the dissociation rate of open complexes [18,19].

In crystal structures [8,12,14,16], the active site cleft is ~70 Å deep and 100 Å long, but only ~15 Å wide. Early cryo-EM structures [48] and a recent FRET study in solution [49] reveal that the cleft is dynamic, with a significant fraction of holoenzyme molecules displaying a much wider (20–25 Å) cleft. This flexibility is likely due to the movement of comparatively rigid RNAP “modules” relative to each other. Multisubunit RNAPs contain five “switch” regions [39,40], which undergo conformational changes during open complex formation, altering relative positions of the modules. Wider clefts, resulting largely from different positioning of the “clamp” module, have been observed in a paused complex [50] and a complex with transcription factor Gfh1 [51]. Opening of the clamp has been proposed to underlie the mechanism of action by the stress alarmone ppGpp [52,53]. Several antibiotics inhibit modular movements of RNAP by binding to or near these switch regions [54,55,56]. 

Opening of the RNAP clamp likely permits loading of the duplex DNA during initiation. Since the hydrated DNA duplex is about 25 Å wide, early models of open complex formation proposed that DNA opening occurred outside the cleft before entry of the strands [13,37,57]. However, the extended downstream footprint (to +20, relative to the transcription start site) observed for advanced closed complexes demonstrates that the downstream duplex enters the active site cleft before it is opened [27,58,59,60]. A more open cleft obviates the need for separating the strands prior to entry. 

## 3. *E. coli* σ^70^ Promoter Recognition

*In vivo*, initiation rates vary at least 10,000-fold for different promoters [61,62]. Rates of open complex formation (at a specified [RNAP]) and dissociation *in vitro* both span a similar range determined by the sequence and structure of the promoter DNA [61,62]. For a given promoter sequence, changes in temperature, salt, and solute concentrations [24,63,64,65,66], as well as additions of protein factors and ligands, can affect these kinetics by 10–1000-fold or more.

Promoter elements have been defined structurally, genetically, and/or functionally (summarized in Figure 1). What promoter regions are most important for which steps in the mechanism? As an extension of the bipartite proposal of promoter function [67], a working hypothesis is that promoter sequence and structure upstream of (and including at least part of) the −10 element direct the steps of initial binding of RNAP and subsequent conformational changes that culminate in bending of the downstream duplex into the cleft. These steps precede the central DNA opening step, which opens approximately 13 bp (−11 to +2 at λP_R_) of the promoter DNA. Collectively these steps determine the rate of open complex formation. For some (but not all) promoters, promoter elements in and downstream of the −10 element direct post-opening steps of the mechanism (in particular open complex stabilization) that determine the lifetime of the open complex. All of these steps are discussed in subsequent sections.

The farthest upstream sequence-specific interactions between RNAP and promoter DNA are in the UP element region from approximately base −40 to −60 (Figure 1, cyan; reviewed in [68]). UP elements are phased AT-tracts recognized by the C-terminal domains of the α subunits (αCTDs); the narrow minor groove at these sequences favors specific αCTD binding [69]. UP elements were first noted for their ability to increase transcription from the rRNA promoters, and a consensus UP element sequence determined using mutational analysis increased transcription 330-fold *in vivo* [70,71]. The entire UP element consists of two subsites, proximal and distal, one for each αCTD; promoters may have one or both, although the distal UP element tends to function nearly as well as a full UP element [72]. Because of the curvature generally observed at phased A- or T-tracts, it was hypothesized that the main function of UP elements was to bend the DNA. However, not all UP elements display significant curvature [73] as even single base disruptions in an A- or T-tract can practically abolish bending [74].

At λP_R_ and T7A1, real time and low temperature DNase and hydroxyl radical (OH) footprinting of closed complexes reveals that recognition of the −35, −10, and UP elements induces bending of upstream DNA at −38 and −48 [27,59,75] and wrapping of the region from ~ −60 to −80 on RNAP [25,26,27,59,75,76]. Structural evidence for bending at the −35 element is provided by [43,77], and a discussion of functional bending by the αCTDs is provided in [68]. These upstream interactions are very important for efficient open complex formation at λP_R_ and *lac*UV5 (see below) [75,78].

The −35 element (Figure 1, blue) has the consensus sequence 5'-TTGACA-3' [79] with the −35T, −34T, and −33G being the most highly conserved [80]. These bases interact via the major groove with a helix-turn-helix motif of σ_4.2_ (Figure 1 and Figure 2, blue), which bends the DNA approximately 36° in cocrystal structures of the −35 element and *Thermus aquaticus* σ_4.2_ [13,43] and a recent initiation complex structure with *E. coli* holoenzyme [77]. Strong DNase I footprinting enhancements suggestive of bending are also observed at the upstream end of the −35 element at λP_R_ [75]. This strong bend observed in solution may be due to σ_4_ interacting with the αCTDs [12,81]. At some promoters, σ_4_ interacts with activators such as λCI [82] and CAP [11,83,84,85], influencing multiple steps of transcription initiation. This region can also interact with the core RNAP to mediate recruitment of elongation factors [86].

There is no consensus sequence for the majority of the spacer between the −35 and −10 elements, but there is a consensus length, which is dictated largely by the spacing between σ_4.2_ and σ_2.3_ [13]. The most common spacer length for σ^70^ promoters is 17 bp [79,87,88], and promoters with 17 bp spacers produce greater amounts of transcript in multi-round assays than otherwise identical promoters [89,90,91]. The length and extent of bending (as predicted by molecular modeling) of the spacer region modulate the effect of σ_1.1_ on transcription initiation kinetics and the structure of the open complex [92]; the underlying mechanism is unknown, but the authors speculate that this may be due to σ_1.1_ acting as a “gatekeeper”, discriminating between promoter and non-promoter DNA based on the trajectory of the spacer from the −35 element to the −10. Recent structures of *E. coli* σ^70^ initiation complexes formed with 4–5 nt nascent RNAs reveal that rotation of σ_4_ is required to accommodate different promoter spacer lengths [77]. A region within the spacer defined as the “Z-element” (bases −24 to −18) was also identified in a recent study as interacting with the β' zipper (residues 40–45), increasing the amount of abortive transcription from a synthetic promoter [93]. It is not known, however, whether this interaction is sequence-specific or simply specific to a certain spacer conformation or trajectory.

At some promoters, an “extended −10” sequence (TGn; Figure 1, red) increases activity through specific contacts with σ_3.0_ [44,94] (Figure 1 and Figure 2, red), perhaps by increasing the lifetime of the open complex [95]. The TGn motif was found in 20% of the 554 promoters identified in one study [87], with 44% of these promoters having a G at −14. The TGn motif may be particularly important at promoters with weaker −35 elements [43] or longer spacers [87]. In *E. coli* initiation complex structure, the extended −10 element is recognized by the insertion of two perpendicular helices of σ_2_ and σ_3_ into the major groove [77].

The −10 element (Figure 1, yellow), with an all-AT bp consensus sequence (5'-TATAAT-3') constitutes the upstream half of the region opened by RNAP, with opening likely initiated near its upstream end. The transcription bubble forms downstream of base −12 (for λP_R_ and other promoters with six bp discriminators; see Figure 1). This position remains base paired [77,96], with the *E. coli* σ^70^ Q437 likely making a key sequence-specific contact [97] with base −12A of the template strand [10]. After opening, the −10 region of the nontemplate strand interacts with conserved residues of σ, with the nearly invariant bases −11A and −7T in pockets of σ^70^ [10,17,98,99]. Residues of both template and nontemplate strands interact with σ_2.4_ [10,17]. Modeling and biochemical data suggest that these contacts require prior strand separation [10]. It remains unresolved whether recognition of this region requires the DNA to be single-stranded.

The base −11A is thought to nucleate DNA opening; alanine substitutions of aromatic residues in the −11A binding pocket on σ_2.3_ impair open complex formation, but not binding to duplex DNA [100]. Flipping of base −11A into its pocket may thus nucleate bending of the DNA into the cleft before opening at some promoters; alternatively, other RNAP-DNA interactions may first bend the DNA, nucleating −11A flipping [10]. A recent comparative kinetic study of all 4096 −10 variants [22] finds that RNAP interactions with −7T play a relatively stronger role in open complex formation kinetics, suggesting a model in which bubble nucleation occurs at −7T, or −7T flipping stabilizes the bubble in the transition state, or both. While −11A and −7T are the most conserved bases of the −10 element [79,88,101], they are not absolutely required for relatively fast initiation kinetics, particularly if the −10 element is sufficiently AT-rich [22]. A recent structure shows that *E. coli* σ^E^ uses a similar base flipping mechanism to recognize, and possibly nucleate opening at, the −10 element [102].

The discriminator region between the −10 element and the start site (Figure 1, orange) is involved in regulation of open complex lifetime. Its upstream end interacts with σ_1.2_ [9,17,77,95,103] (Figure 1 and Figure 2, orange). Most discriminators are 6–8 bases in length [88]. Mutational analyses [104,105,106] indicate that lifetime decreases as discriminator length increases from 6 to 8 bases. A bioinformatic analysis of *E. coli* promoters [107] found that the frequency of discriminators also decreases with increasing length from 6 to 8 bases. These observations together indicate that many *E. coli* promoters form long-lived open complexes. More recent efforts to comprehensively map the transcription start sites in *E. coli* [108] will no doubt lead to elucidation of the *in vivo* importance of the sequence of this region. Structural data indicate that for a six-base discriminator, −6G on the nontemplate strand flips into a pocket on the surface of σ_1.2_ [17]. The −5 nontemplate strand base is also very important for open complex stability, with a G being relatively stabilizing and a C being relatively destabilizing [95]. Similarly, σ^A^ from *T. aquaticus* binds most favorably to a 5'-GGG-3' sequence immediately downstream of the −10 element [109]. The rRNA promoters generally have a C on the nontemplate strand two bases downstream of the −10 element [110], leading to short open complex lifetimes; this sequence element is important for their regulation *in vitro* and *in vivo* [95].

A recent crystal structure of a model open complex identifies a “core recognition element” (or “CRE”, bases −4 to +2 on the nontemplate strand) recognized by the β subunit [17]. Base +2G is flipped into a pocket, increasing the lifetime of an RNAP-fork junction complex containing a consensus −10 element and strong discriminator [17]. Each CRE base except −1 appears to be specifically recognized, and substitutions in the corresponding β residues lead to defects in transcription initiation. No consensus sequence of CRE has been detected [87] and thymine bases in this region are generally observed to be reactive to permanganate, implying that they are exposed to solvent. However, *in vivo* footprinting argues that CRE interacts with RNAP during elongation [111] and base-specific interactions involving the CRE have been implicated in pausing [112].

The most common transcription start site base is A ≥ G > T >> C [106]. Subsaturating concentrations of the initiating nucleotides affect the kinetics of initiation, just as the concentration of any substrate affects the velocity V of an enzyme-catalyzed reaction for subsaturating concentrations (where V < V^max^). In addition, binding of initiating NTP stabilizes the short-lived open complex at rRNA promoters [113], shifting the distribution of promoter complexes from closed to open in a NTP concentration-dependent manner.

## 4. Initial Binding and Subsequent Conformational Changes to Form and Stabilize the Open Complex at Eσ^70^ Promoters

**Figure 3:**
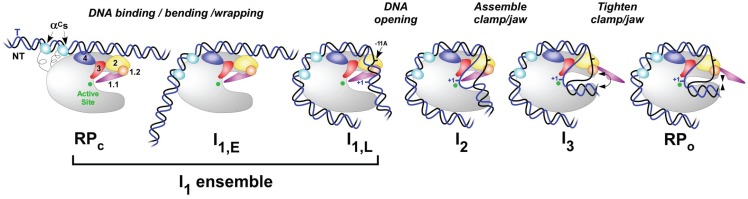
Schematic representation of proposed intermediates in open complex formation and dissociation at λP_R_ promoter. Closed complexes like RP_C_, I_1,E_ (early), or I_1,L_ (late) can be significant members of the rapidly equilibrating I_1_ ensemble. The αCTDs are shown in cyan; other colors are the same as in Figure 1.

Ensemble and single-molecule kinetic-mechanistic studies with wild-type or variant RNAP and promoter DNA, DNA footprinting in real time or at low temperature, crosslinking and fluorescence studies, and high resolution structures of initial and final states provide much information about the sequences of conformational changes and intermediate complexes on the pathway that forms the initial open complex and converts it (at some but not all promoters) to a more stable, longer-lived open complex (Figure 3). Initial specific binding to the promoter sets in motion conformational changes in which the RNAP molecular machine operates on promoter DNA to bend, wrap, and open the duplex and stabilize the open complex, with mobile regions of RNAP playing key roles. The rates and equilibria of these conformational changes are functions of promoter sequence, solution conditions, and regulatory factors or ligands. Rates of open complex formation and lifetimes of the most stable open complex differ by three to four orders of magnitude or more for different promoters under typical *in vitro* transcription conditions. In this section, we qualitatively discuss this pathway. In the following section, we discuss the interpretation of the experimentally determined rate and equilibrium constants for open complex formation and dissociation in terms of these conformational changes.

### 4.1. The Promoter Search and Initial Specific Binding to Form RP_C_

During initiation, RNAP holoenzyme first searches for and specifically recognizes promoter DNA, forming an initial closed complex generally called RP_C_ (Figure 3). The bimolecular rate constant for formation of a closed complex between Eσ^70^ and *rrnB* P1 promoter on a short (204 bp) DNA is 2 × 10^8^ M^−1^·s^−1^ [71]; that for a closed complex between Eσ^54^ RNAP and an 853 bp fragment containing the *S. typhimurium glnAp2* promoter is 2.1 × 10^7^ M^−1^·s^−1^ [21]. These provide lower bounds on the rate constant for RP_C_ formation at these promoters, which are one or two orders of magnitude less than the theoretical three-dimensional (3D) diffusion limit (~6 × 10^9^ M^−1^·s^−1^ for equal-sized reactants). Is the rate of formation of the initial specific complex RP_C_ determined by 3D diffusion of RNAP and promoter DNA, or by nonpromoter binding followed by 1D diffusion (*i.e.*, sliding) and/or hopping of RNAP which can increase the rate constant above the 3D limit? Although some early studies (including [114,115,116,117,118]) argued in favor of RNAP sliding, recent single-molecule studies provide evidence against it [119,120], while suggesting a role for RNAP hopping [121]. Since thermodynamics is path-independent, sliding or hopping should not affect the equilibrium constant for forming RP_C_. This equilibrium constant and not the second order rate constant for forming RP_C_ is generally the significant quantity for the kinetics of open complex formation (see Section 5). Hence sliding or hopping (if they occur) will increase the rate of open complex formation and initiation only if RP_C_ formation is irreversible. Nonpromoter binding of RNAP [122] can have either a competitive or facilitating effect on promoter binding, depending on DNA length and solution conditions. 

The initial specific closed complex RP_C_ (Figure 3) and/or other early specific promoter complexes have been characterized by real time OH and permanganate footprinting of the T7A1 promoter [25,26] and by low temperature DNase, OH and permanganate footprinting of other promoters [58,123,124,125,126,127]. Footprinting reveals that RP_C_ is closed and that RNAP is bound to the UP element, −35, spacer and −10 regions, protecting the upstream DNA from −55 to −5. Since no sites of enhanced DNase reactivity are observed in RP_C_s [58,123,124,125,126,127] the downstream duplex is presumably unbent. At λP_R_, only more advanced closed complexes than RP_C_ have been detected, indicating that RP_C_ must be significantly less stable than these extended-footprint closed complexes under the conditions studied.

### 4.2. Upstream Bending and Wrapping to Form More Advanced Closed Complexes 

The first conformational changes induced by RP_C_ formation may occur upstream of the −35 region. DNase footprinting of λP_R_ closed complexes reveals enhanced reactivity at −38 on the template strand and within the UP element region (−48 on the template strand, −45 on the nontemplate strand), indicating DNA bending or other distortions at these positions [75]. Periodic protection of the upstream DNA from OH attack is observed upstream to approximately base −80 [27], and downstream to base +20. This indicates that the far upstream promoter duplex is lying on the “back” surface of RNAP (see Figure 3 and [27]).

The mechanism of forming this bent, wrapped interface is unclear. Since the αCTD are on flexible tethers, extension of the tethers allows them to bind specifically to the UP element or nonspecifically to DNA upstream of the −35 element in RP_C_ (see Figure 3). Specific binding to the proximal UP element brings one αCTD (binding site centered at −42) into contact with region 4 of σ^70^; mutation of σ region 4 residues within this σ_4_-αCTD interface reduces transcription from some, but not all, UP element-containing promoters [81,83]. A second αCTD binds to the distal site, centered at base −52 [72]. There is no evidence that the two αCTD interact in open complex formation [68], but efficient transcription from promoters having only a distal UP element requires both αCTDs, suggesting that the second αCTD must somehow affect the overall stability of the complex [68,72]. In the model of Davis *et al.* [27], formation of these interfaces bends the DNA between −35 and −60 nearly 100°, driving presumably nonspecific interactions between RNAP and DNA upstream of base −60.

Interactions of the upstream promoter DNA with the αCTD are observed to be very important for efficient open complex formation at λP_R_, which appears to have a distal UP element [75], and for efficient initiation at *lac*UV5, which has no UP element [78]. Truncation of λP_R_ at base −47 (removing the specific distal UP element sequence and upstream DNA and, thus, probably most of the effect of the UP element [72]), or of *lac*UV5 at base −45 reduces the rate of the isomerization/DNA opening step (see Section 5) by 1.5–2 orders of magnitude; deletion of the αCTD and mutation of a single residue which eliminates αCTD-DNA binding have similar effects [78,128]. Smaller but significant effects of truncations upstream of −60 on the isomerization/DNA opening step are also observed [78,128]. Because the strong dependences of the kinetics on upstream DNA length are similar for both promoters, only nonspecific interactions with the αCTD appear necessary to generate these effects.

Far upstream interactions persist in the open complex at some (perhaps many) promoters. For *lac*UV5 open complexes, crosslinks between the αCTDs and nonspecific upstream DNA are observed even to base −90 [129]. Atomic force microscopy demonstrates that at λP_R_, DNA is extensively wrapped around RNAP in the open complex [76].

### 4.3. Bending the Downstream Duplex into the Cleft

In RP_C_, the downstream duplex (−5 to +20) is unprotected from OH and DNase, indicating that it is not interacting with the RNAP cleft [58,123,124,125,126,127]. By contrast, at λP_R_, the OH footprint of an advanced closed complex (like I_1,L_ in Figure 3, obtained in real time [27]) is periodic and extends from −80 to +20, indicating that one face of the downstream duplex is in the cleft or otherwise protected by RNAP. Closed complexes with similar downstream footprint boundaries (between +15 and +25) are observed at other promoters and have been variously called RP_i_ (at *rrnB* P1 [130,131], *lac*UV5 [127], and T7A1 [126]), RP_C_ (at *λprmup-1* Δ265[132]) and RP_C2_ (at the σ^32^ promoter *groE* [58,125]).

In the conversion of RP_C_ to I_1,L_ (Figure 3) the downstream DNA must be bent by at least 90° at the upstream end of the −10 element to enter the cleft [16,60]. What bends the duplex is not known. The −11A base flips out of the stacked duplex and into a pocket of σ^70^ region 2 [9,10,17]. If base flipping occurs before this region is bent, it would introduce a point of greater flexibility into the duplex, resulting in spontaneous bending of the duplex and its capture by the cleft. Alternatively, bending of the −10 region by RNAP may occur first, destabilizing the stacking and resulting in base flipping. If the interaction of σ^70^ region 2 with the unbent −10 region in early closed complexes were limited to the −12 position, this interaction might either induce flipping of the adjacent −11A base or dictate the site of bending and subsequent base flipping after a downstream interaction [10,97].

In-cleft elements like σ_1.1_ and downstream mobile elements (DME; see below) of free RNAP holoenzyme are positioned (Figure 2) to prevent entry of nonpromoter DNA into the active site cleft. These elements of RNAP appear to be part of an extensive, sophisticated network of upstream and downstream interactions that regulate the access of the downstream duplex to the cleft to form I_1,L_. Conversion of RP_C_ to I_1,L_ appears to involve a series of closed intermediates with different extents of downstream interaction, in which the extent of bending into the cleft may be coupled to the extent of upstream bending and wrapping discussed above. This regulatory network responds to promoter sequence and structure, as well as factor binding and solution conditions. Many of these same elements may also be involved in the regulation of open complex lifetime (see below).

Several lines of evidence exist for this network. As discussed above, truncation of upstream DNA greatly reduces the rate of the isomerization step of open complex formation. In addition to this, truncation of λP_R_ at −47 shortens the downstream DNase footprint boundary of the advanced closed complex ensemble from +20 to +7 on the nontemplate strand and +2 on the template strand, indicating that only the upstream half of the downstream duplex (−10 to +2/+7) is inserted in the cleft in this closed complex [75]. Similarly, a RNAP variant lacking σ_1.1_ forms a closed complex footprint at early times at λP_R_, with a downstream boundary of ~ −5 [47]. Thus, upstream DNA interactions and σ_1.1_ facilitate bending of the downstream duplex into the cleft.

Similarly, at *rrnB* P1, the complex formed at equilibrium in the absence of nucleotides at 12 °C has a downstream boundary which is approximately +1 [130,131]. This footprint boundary requires that there be some bending at the −10 element, but not enough to fully load bases −10 to +20 into the cleft. What could be the reason for this partial bending in these complexes? We propose that impediments (σ_1.1_ and/or DME) to full entry of the downstream duplex into the cleft have not been fully removed.

At λP_R_, the series of conformational changes initiated by the binding interactions in RP_C_ and culminating with bending the downstream duplex fully into the active site cleft are prerequisite for subsequent efficient DNA opening [27,64]. Upstream bending and wrapping are clearly targets of regulation by factors and ligands. Some transcription factors (e.g., CAP, IHF, Fis, and other nucleoid associated proteins) bind in or near the −40 to −60 region, bending DNA while also replacing or affecting the interaction of this region with the αCTD [6,7,68,133].

### 4.4. DNA Opening and Closing in the Cleft

**Figure 4:**
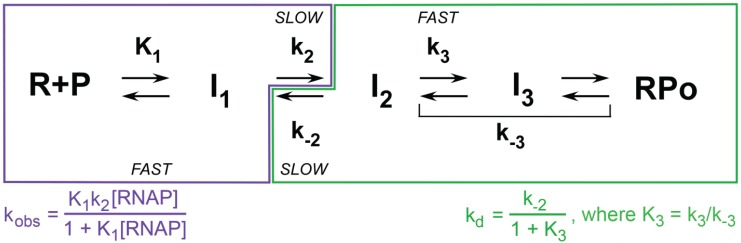
Mechanism of transcription initiation: resolvable kinetic steps. Minimal mechanisms of open complex formation and dissociation, showing by color coding the steps that contribute to the observed rate constants (k_obs_, k_d_) for promoters like λP_R_ that form a stable open complex RP_O_. For the common experimental situation of excess RNAP, the rate constant for open complex formation (k_obs_) is determined by the front half of the mechanism (boxed in purple), including the equilibrium constant K_1_ for formation of the ensemble of closed (I_1_) intermediates, the forward rate constant k_2_ of the isomerization step that includes DNA opening, and the excess RNAP concentration. Likewise, k_d_ is determined by the late steps of the mechanism (boxed in green), including the rate constant k_-2_ for DNA closing and the equilibrium constant K_3_ for stabilization of the initial open complex I_2_ to form longer-lived I_3_ and/or RP_O_ complexes [65,66].

At λP_R_ opening of the entire 13 bp transcription bubble occurs in the rate-determining isomerization step that forms the first open intermediate, I_2_ (k_2_ in Figure 4) [23]. As for DNA melting in solution in the absence of RNAP, this step is highly temperature dependent, increasing 1000-fold between 7 °C and 42 °C with an activation energy of 34 kcal [60,66]. However, unlike in solution, the DNA opening step on RNAP is only weakly dependent on the concentrations of salt and solutes, consistent with a scenario in which it occurs in the local environment of the cleft [64]. The process of transcription bubble closing (k_-2_ in Figure 4), which is rate determining in dissociation, is even less affected by salt and solute concentration [24] and, at λP_R_ and T7A1, does not appear to be strongly affected by DNA sequence or any modification of RNAP tested thus far, including large deletions [18].

Because the rate and equilibrium constants for transcription bubble opening and closing are only weakly (if at all) dependent on salt and solute concentrations, we propose that the regulation of the rate of open complex formation (and the rate of initiation in cases when open complex formation is the bottleneck) occurs primarily in the steps that form and remodel the closed complex, but not in the actual DNA opening step. However, whether this mechanism is universal is still a subject of some debate. Comparison of the timing of permanganate reactivity and downstream DNA protection at T7A1 has led to the proposal that at T7A1 opening occurs before the DNA is bent into the active site cleft [25].

### 4.5. Open Complex Stabilization

The conversion of the relatively unstable initial open complex (I_2_) to the highly stable open complex (RP_O_) at λP_R_ involves conformational changes in DNA and RNAP. Comparison of permanganate footprints of λP_R_ I_2_ and RP_O_ indicates that the discriminator region of the nontemplate strand is repositioned in the cleft, becoming more reactive in RP_O_ [23,95]. The large dependences of the equilibrium constant K_3_ for this step on urea and salt concentration indicate the folding/assembly of approximately 120 RNAP residues of the jaw and other DME on the downstream duplex [134]. Comparative quantitative studies with λP_R_ truncated at base +12 and a β' jaw deletion mutant of RNAP indicate that the β' jaw DME [127] (Figure 2, brown) and β' sequence insertion 3 DME (also termed β' insertion 6; Figure 2, green) are involved in the assembly-DNA binding process that converts I_2_ to the much more stable RP_O_ at λP_R_. In this model, supported by studies using RNAP and DNA heteroduplexes of varying lengths [29,30,31,135], the DME assemble on downstream DNA, stabilizing the open complex. Recent evidence suggests that the interaction of DNA between positions +4 and +6 with switch regions 1 and 2 may trigger cleft closure and DME folding/assembly and clamping [29].

Kinetic dissection of open complex stabilization at λP_R_ led to the discovery of a third on-pathway intermediate designated I_3_ [24]. Formation of I_3_ from I_2_ involves the majority (approximately two-thirds) of the folding and assembly of RNAP DME involved in converting I_2_ to RP_O_. The extent of this assembly is intriguingly similar to that observed for the open complex formed by a β' jaw deletion variant, which may therefore be an analog of I_3_ [134]. Another potential analog, based on similarity in lifetime and downstream interactions, might be the open complex formed at T7A1 [18,136]. The lifetime of the open complex formed with *rrnB* P1 (~1 s) [95] is intriguingly similar to that of I_2_ [24], consistent with the idea that the *rrnB* P1 sequence (in particular the weak discriminator and short spacer) precludes the stabilizing interaction from cleft closing and DME assembly induced by promoters like λP_R_ and T7A1. Further experiments are required to explore the roles of promoter elements in the mechanism, and the potential relevance of I_2_, I_3_ and other forms of the open complex to transcription initiation and its control.

## 5. Forming and Stabilizing the Open Complex; Regulation of the Kinetics by Promoter Sequence, Length and Other Variables

Kinetic studies are essential to any determination of mechanism, together with structural information about intermediates. Among other insights, kinetic data provide evidence for the minimum number of key intermediates and indicate how to trap or populate them. Knowledge of kinetics and mechanism is necessary to understand how promoter sequence, regulatory factors, ligands, and solutes/salts exert their effects, and to design inhibitors, including antibiotics.

### 5.1. Kinetics and Mechanism of Open Complex Formation

The kinetics of open complex formation in excess RNAP at concentration [R] are found to be single exponential with a rate constant (k_obs_), which is a hyperbolic function of [R]. These kinetic data are well described by Mechanism 1 (see also Figure 4) with a reversible initial step (with equilibrium constant K_1_) that includes promoter binding and forms a closed intermediate I_1_, followed by a rate determining “isomerization” step (with rate constant k_2_) to convert I_1_ to the stable open complex (RP_O_) [137,138].

Mechanism 1 $${\text{K}}_{1}\;\;\;\;\;\;{\text{k}}_{2}\;\text{R}+\text{P}⇄{\text{I}}_{1}\to \text{R}{\text{P}}_{\text{O}}$$

While evidence now exists for an ensemble of closed intermediates I_1_ and several different intermediate open complexes, Mechanism 1 remains useful for analysis of the kinetics of open complex formation, and the quantities K_1_ and k_2_ are interpretable in terms of the species in the I_1_ ensemble.

The observation of single exponential kinetics means that the species in the I_1_ ensemble rapidly equilibrate with one another and with free P on the time scale of its conversion to RP_O_ [139]. Analysis of Mechanism 1 for this situation (see Figure 4, bottom) yields:

k_obs_ = θ_I1_k_2_ (2)

where θ_I1_ is the fraction of closed promoter DNA present as I_1_ complexes:

(2) $${\text{θ}}_{\text{I}1}=\frac{{\text{[I}}_{\text{1}}\text{]}}{\left[\text{P}\right]{\text{ + [I}}_{\text{1}}\text{] }}=\frac{{\text{K}}_{\text{1}}\left[\text{R}\right]}{{\text{1 + K}}_{\text{1}}\left[\text{R}\right]}$$

The interpretation of K_1_ and k_2_ depends on the details of the I_1_ ensemble, as indicated for the particular case of two closed complexes below.

### 5.2. Kinetics of Open Complex Formation for λP_R_ and λP_R_ Variants: Interpretation of k_obs_

As an example relevant for analysis of the single-exponential kinetics of open complex formation by full-length (FL) and upstream truncated (UT) λP_R_ promoters, consider the situation where the I_1_ ensemble consists of one early closed complex (designated I_1,E_) and the most advanced closed complex (I_1,L_). Since the kinetics of RP_O_ formation are single-exponential, I_1,E_ and I_1,L_ are in rapid equilibrium with one another and with P on the time scale of conversion of I_1,L_ to RP_O_. For this case Mechanism 1 is rewritten as:

Mechanism 2 $${\text{K}}_{\text{1,E}}\;\;\;\;\;\;{\text{K}}_{\text{1,L}}\;\;\;\;\;\;{\text{k}}_{\mathrm{open}}\;\text{R}+\text{P}⇄{\text{I}}_{\text{1,E}}⇄{\text{I}}_{\text{1,L}}\to \text{R}{\text{P}}_{\text{O}}$$

where k_open_ is the rate constant for the elementary, rate-determining DNA opening step involving I_1,L_.

For Mechanism 2, k_obs_ in excess RNAP is given by Equation (3):

k_obs_ = θ_I1L_k_open_ (3)

where θ_I1L_ is the fraction of closed promoter DNA present as I_1,L_ complexes:

(4) $${\text{θ}}_{\text{I1L}}=\frac{{\text{I}}_{\text{1,L}}}{{\text{P+ I}}_{\text{1,E}}{\text{ + I}}_{\text{1,L}}}=\frac{{\text{K}}_{\text{1,E}}{\text{K}}_{\text{1,L}}\text{[R]}}{{\text{1+ K}}_{\text{1,E}}{\text{(1+ K}}_{\text{1,L}}\text{)[R]}}$$

Comparison of Equations (1)–(4) shows that the observed isomerization rate constant k_2_ of Mechanism 1 is interpreted using Mechanism 2 as:

(5) $${\text{k}}_{2}=\frac{{\text{K}}_{\text{1,L}}}{{\text{1+ K}}_{\text{1,L}}}{\text{k}}_{\text{open}}={\text{f}}_{\text{I1,L}}{\text{k}}_{\text{open}}$$

where ${\text{f}}_{\text{I1,L}}$ is the fraction of I_1_ complexes that are I_1,L_:

(6) $${\text{f}}_{\text{I1,L}}=\frac{{\text{K}}_{\text{1,L}}}{{\text{1+ K}}_{\text{1,L}}}=\frac{{\text{[I}}_{\text{1,L}}\text{]}}{{\text{[I}}_{\text{1,E}}{\text{] + [I}}_{\text{1,L}}\text{]}}$$

The observed closed complex binding constant K_1_ of Mechanism 1 is interpreted using Mechanism 2 as:

(7) $${\text{K}}_{1}={\text{K}}_{\text{1,E}}\;(1+{\text{K}}_{\text{1,L}})=\frac{{\text{[I}}_{\text{1,E}}{\text{] + [I}}_{\text{1,L}}\text{]}}{\text{[P][R]}}$$

In words, K_1_ of Mechanism 1 is the overall I_1_ binding constant written in terms of total I_1_ concentration. If the mechanism is more complicated than Mechanism 2, with additional on-pathway closed complexes in rapid equilibrium with one another on the time scale of DNA opening, Equations (5) and (7) increase in complexity, but the general principle still applies that the observed isomerization rate constant k_2_ is the product of k_open_ and the fraction of closed complexes that are I_1,L_. Only if all closed complexes are I_1,L_ (*i.e.*, K_1,L_ >> 1) does k_2_ = k_open_; in this case K_1_ = K_1,E_K_1,L_ for Mechanism 2.

**Figure 5:**
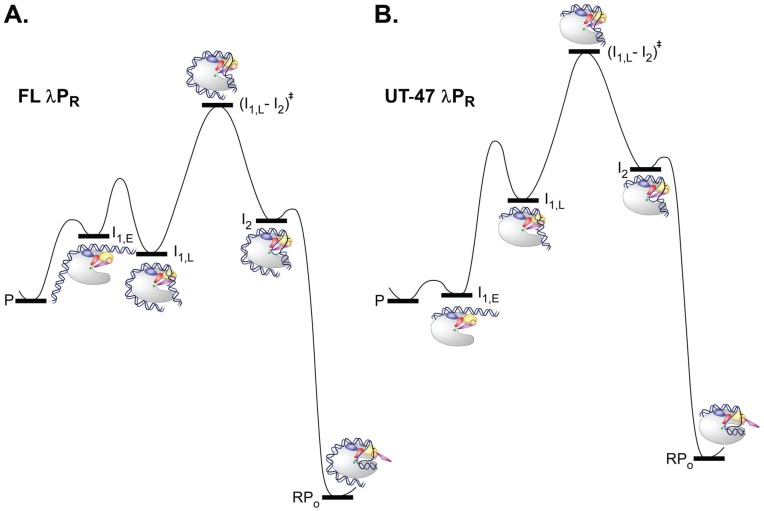
Reaction progress diagrams for open complex formation by (**A**) FL and (**B**) UT−47 λP_R_, interpreted using Mechanism 2. Free energies of the rapidly-equilibrating closed intermediates I_1,E_ and I_1,L_, the rate- determining transition state (I_1,L_-I_2_)^‡^, and the initial (I_2_) and stable (RP_O_) open complexes are calculated relative to promoter DNA (P) for [R] = 30 nM from experimentally-determined values of K_1_, k_2_, k_-2_ and k_d_ for FL λP_R_ [24,60] and UT-47 λP_R_ [75] at 37 °C (see text for details). Free energies of FL and UT-47 λP_R_ are set at the same value. For purposes of illustration, for both variants, we estimate that k_open_ = 1 s^−1^ and that (I_1,L_-I_2_)^‡^ decomposition frequencies are 10^3^ s^−1^ in each direction, and assume that k_-2_ for UT-47 λP_R_ is the same as that determined for FL λP_R_ [24].

Here we apply Mechanism 2 to interpret the large differences in K_1_ and especially in k_2_ for open complex formation by full length (FL) and upstream-truncated UT-47 λP_R_ promoters at 37 °C [75]. Isomerization is much faster for FL λP_R_ (k_2_ = 0.66 s^−1^) than for UT-47 λP_R_ (k_2_ = 0.03 s^−1^), but closed complex binding is weaker (K_1_ = 5.8 × 10^6^ M^−1^ for FL; K_1_ = 2.6 × 10^7^ M^−1^ for UT-47.) For FL λP_R_, low temperature equilibrium OH footprinting [59] and fast OH footprinting [140,141] reveal that the population of closed I_1_ intermediates is advanced with the DNA significantly protected to +20. Hence the observed isomerization rate constant k_2_ is probably not much less than the DNA opening rate constant k_open_. Here, for purposes of illustration, we estimate k_open_ to be 1 s^−1^ at 37 °C. (The maximum rate of transcription initiation at 37 °C is known to be ~ 1 s^−1^ [61,62], indicating that k_open_ ≥ 1 s^−1^.) From k_2_/k_open_ = 0.66 and Equation (5), K_1,L_ = 2 so 2/3 of closed complexes are I_1,L_, consistent with the footprinting data on the I_1_ ensemble. From K_1_ = 5.8 × 10^6^ M^−1^ and the interpretation of k_2_ in Equation (5) and Mechanism 2, K_1,E_ = K_1_/3 = 1.9 × 10^6^ M^−1^. For FL λP_R_ a plausible free energy *vs.* progress diagram for the steps of open complex formation is given in Figure 5A, which is drawn to scale for [R] = 30 nM, corresponding to K_1,E_[R] = 0.06 and a correspondingly small occupancy of I_1,E_ at 30 nM RNAP. The barrier height for the rate determining opening step (rate constant k_open_ = 1 s^−1^) is set for (I_1,L_-I_2_)^‡^ transition state decomposition frequencies of 10^3^ s^−1^ in each direction [128].

For the UT-47 λP_R_ variant, in which the distal UP element and far upstream DNA are missing, k_2_ is only 3% of the FL λP_R_ value [75], even though there is no obvious reason for upstream truncation to affect the intrinsic opening rate constant k_open_, and the closed complex binding constant K_1_ is 4.5 fold larger than for FL λP_R_, even though favorable distal UP element interactions are eliminated by truncation at −47. Should the large reduction in k_2_ for UT-47 be interpreted as a reduction in k_open_ or as a shift in the closed complex ensemble to less advanced species? Footprinting evidence supports the latter interpretation: in UT-47 I_1_ ensemble, the downstream boundary of DNase protection is at +2/+7, indicating that the downstream duplex is only partially bent into the cleft. Hence, the absence of far-upstream DNA results in a less advanced closed complex ensemble for UT-47 λP_R_.

At 37 °C, interpretation of k_2_ = 0.02 s^−1^ for UT-47 λP_R_ [75] in terms of Mechanism 2 with k_open_ = 1 s^−1^ indicates that K_1,L_ ~ 0.02, indicating that only 2% of the population of closed complexes are I_1,L_ and 98% are I_1,E_, very different from the 66% I_1,L_, 34% I_1,E_ distribution for FL λP_R_. From K_1_ = 2.6 × 10^7^ M^−1^ [75] and this interpretation of k_2_, K_1,E_ = K_1_/1.02 = 2.5 × 10^7^ M^−1^, 13-fold larger than that calculated for FL λP_R_. The free energy vs progress diagram for the steps of open complex formation with UT-47 λP_R_ is given in Figure 5B, which is also drawn to scale for an excess RNAP concentration of 30 nM, corresponding to K_1,E_[R] = 0.76 and a much higher occupancy of I_1,E_, relative to both free promoter DNA and I_1,L_, than for FL λP_R_. As in Figure 5A, the barrier height for the rate determining opening step (rate constant k_open_ = 1 s^−1^) is set for a transition state decomposition frequency of 10^3^ s^−1^ in each direction.

For UT-47, formation of I_1,L_, required for the subsequent opening step, is greatly disfavored by upstream truncation of the promoter DNA, resulting in a much reduced isomerization rate even though the intrinsic opening rate k_open_ is left unchanged. The very significant increases in K_1_ (and K_1,E_ in Figure 5B) for UT-47 relative to FL λP_R_ are difficult to explain, since they indicate that the far-upstream DNA actually destabilizes early closed intermediates like I_1,E_. Presumably only the αCTD are capable of extending sufficiently to interact with far upstream DNA in I_1,E_, but if such interactions are unfavorable it is unclear why they would occur. Published kinetic data for the effect of deleting σ_1.1_ on the kinetics of open complex formation show similar effects to those of upstream truncation: the isomerization rate constant is dramatically reduced while the closed complex binding constant increases [47]. In this case, deletion of 1.1 may reduce both K_1,L_ and k_open_, while increasing K_1,E_.

### 5.3. Kinetics and Mechanism of Open Complex Dissociation

Dissociation of the stable open complex RP_O_ to free promoter DNA upon addition of a competitor (to make dissociation irreversible) exhibits first order, single-exponential kinetics with rate constant k_d_ (Figure 4). For the two promoters investigated, λP_R_ [65,66] and *lac*UV5 [61], both of which form long-lived RP_O_ complexes at 37 °C, k_d_ decreases strongly with increasing temperature. This negative activation energy of dissociation indicates that one or more intermediates are kinetically significant in dissociation, and (together with the single-exponential kinetics) indicates these intermediates are in rapid equilibrium with RP_O_ on the time scale of the rate-determining step in dissociation. These intermediates occur after the “bottleneck” step in the forward direction and do not contribute to the rate of RP_O_ formation. How many intermediates are there, and are they open or closed? A combination of kinetic and fast permanganate footprinting studies reveal that at least two such intermediates are kinetically significant at λP_R_ (designated I_2_ and I_3_ in Figure 3 and Figure 4); these are open complexes with the bubble extending from −11 to +2 (like RP_O_) which are much less stable than RP_O_ [23,24]. DNA closing occurs in the conversion of I_2_ to I_1,L_ [23]; this step is rate-determining in dissociation [24], as indicated in Figure 5. Though the barrier for conversion of RP_O_ to I_2_ is much larger than that for conversion of I_2_ to I_1_, the rate constant k_-3_ for conversion of RP_O_ to I_2_ does not determine the rate of dissociation because this step is rapidly reversible on the time scale of conversion of I_2_ to I_1_. Hence, the equilibrium constant K_3_ for conversion of RP_O_ to I_2_ and not the rate constant k_-3_ appears in the equation for k_d_ in Figure 4.

Even though more than one open intermediate is involved in dissociation of RP_O_ at λP_R_, kinetic data are well described by Mechanism 3, involving one intermediate open complex (I_2_):

Mechanism 3 $$\frac{\text{1}}{{\text{K}}_{\text{3}}}\;{\text{k}}_{\text{-2}}\;\text{R}{\text{P}}_{\text{O}}⇄{\text{I}}_{2}\to \text{R}+\text{P}$$

The observation of single exponential dissociation kinetics means that I_2_ and any other intermediates like I_3_ rapidly equilibrate with RP_O_ on the time scale of conversion of I_2_ to closed complexes [139]. In other words, in dissociation of RP_O_ without a high salt upshift (see below), any I_2_ formed usually reverts to RP_O_ but occasionally undergoes the rate-determining DNA closing step to form I_1,L_, which rapidly dissociates.

Analysis of Mechanism 3 relates the observed rate constant k_d_ for RP_O_ dissociation to the DNA closing rate constant k_-2_:

k_d_ = f_I2_k_-2_ (8)

where f_I2_ is the fraction of open complexes that are I_2_:

(9) $${\text{f}}_{\text{I2}}=\frac{\text{1}}{{\text{1 + K}}_{\text{3}}\;}\;=\;\frac{{\text{[I}}_{\text{2}}\text{]}}{\left[{\text{I}}_{\text{2}}\right]{\text{ + [RP}}_{\text{O}}\text{] }}$$

To date the intermediate open complex I_3_ at λP_R_ has been treated as part of the RP_O_ population, as shown in Figure 4, because neither the equilibrium constant nor rate constants for forming I_3_ from RP_O_ have been determined [24]. Hence, the equilibrium constant K_3_ in Equation (9) is a composite of those for the conversions of I_2_ to I_3_ and I_3_ to RP_O_.

### 5.4. Determining the DNA Closing Rate Constant (k_-2_) and the Stabilization (K_3_) of the Initial Open Complex 

Fast salt-upshift dissociation experiments provide a valuable method of determining the DNA closing rate constant k_-2_ [24]. For λP_R_ (and presumably other promoters with long-lived open complexes), the stabilization equilibrium constant K_3_ decreases strongly with increasing salt or urea concentration, so a fast salt or urea upshift rapidly converts the initial population of RP_O_ to I_2_ [24]. Because the DNA closing step is found to be independent of salt concentration, a transient burst of I_2_ is obtained, suitable for determining the DNA closing rate constant k_-2_ [24] and for fast DNA footprinting of I_2_ [23]. Moreover, once determined at high salt concentration, k_-2_ is used at lower salt concentrations to dissect k_d_ and determine K_3_ from Equation (9). For λP_R_ at 37 °C, RP_O_ lifetime (1/k_d_) is approximately 11 hours, while that of I_2_ (1/k_-2_) is about 1 second. Therefore, K_3_ is about 10^5^, and RP_O_ is approximately 10^5^ fold more stable than I_2_.

The strong dependences of K_3_ on urea and KCl concentration [18,24] and the effect on K_3_ of deleting the β' jaw or the downstream duplex [134] indicate that a major part of this stabilization involves assembly of the jaw and other DMEs on the downstream duplex, schematically illustrated in Figure 3. Movements of the downstream (discriminator) region of the nontemplate strand [23] and of RNAP elements in the cleft in concert with tightening of interactions in the cleft are also implicated in stabilization of the initial open complex.

### 5.5. Fundamental Similarities of the RNAP-Promoter Mechanism to a Mechanism of Enzyme Catalysis; Implications for Regulation of Open Complex Formation and Lifetime

Mechanism 1 is of course formally the same as the minimal two-step mechanism of enzyme catalysis. The hyperbolic dependence of k_obs_ on [RNAP] in Equation (1) is completely analogous to the hyperbolic dependence of the initial velocity of the enzyme catalyzed reaction on substrate concentration for noncooperative enzymes; K_1_ and k_2_ are the counterparts of 1/K_M_ and k_cat_ in enzyme kinetic analysis. Another fundamental analogy, at least for λP_R_, is that the DNA opening-closing step in mid-mechanism is rate-determining in both directions of the mechanism, just as the central catalytic steps are typically rate determining for both directions of an enzyme catalyzed reaction.

Regulation of the kinetics of enzyme catalyzed reactions by ligands or cooperativity of multi-subunit enzymes is primarily at the level of the initial reversible steps of substrate binding and conformational change that precede the central catalytic step, while the catalytic step itself is typically not regulated. Is this also true of the kinetics of open complex formation and the lifetime of the stable open complex? Both the initial binding step and the conformational changes in the I_1_ ensemble that prepare the DNA to be opened are highly regulated; equilibrium constants for these steps are strong functions of promoter sequence and length [142] and of concentrations of transcription factors, ligands, solutes and salts [24,60,63,64,65,66]. Likewise, the steps converting the initial open complex I_2_ to the stable open complex (RPo at λP_R_) are strong functions of promoter sequence and solute and salt concentrations, and are, thus, also likely targets of regulation.

On the other hand, evidence to date indicates that while the DNA opening and closing rates are strongly temperature dependent (especially DNA opening), these analogs of the catalytic step are relatively insensitive to solution variables. For example, closing rate constants k_-2_ are moderately temperature dependent [24] but only weakly dependent on promoter sequence, and are similar for WT and deletion variant RNAP lacking σ_1.1_ and/or DME regions. Isomerization rate constant k_2_ for λP_R_, thought to be a close approximation to k_open_ (see above), is strongly temperature dependent [60] but is independent of urea concentration and is only weakly salt concentration dependent [24,60,63,64]. We therefore propose that the central DNA opening-closing step is relatively universal and unregulated for Eσ^70^ promoters, and always involves interconverting the most advanced closed complex and the initial open complex. Sophisticated networks of interactions and conformational changes, directed by promoter sequence elements, determine the equilibrium constants of the steps that bracket the central DNA opening-closing and thereby regulate the rate of open complex formation and open complex lifetime.

## 6. Conclusions

In this review, we summarize evidence for the series of large conformational changes, set in motion by binding of bacterial RNAP to promoter DNA to form the initial closed complex and involving bending and wrapping of upstream DNA, which allow the downstream duplex DNA to be bent into the active site cleft of RNAP to form an advanced closed complex. Studies of full-length and upstream truncated promoters indicate that formation of this advanced closed complex is necessary for the subsequent bottleneck step in which the transcription bubble is opened using binding free energy, placing the template strand in the RNAP active site and forming an initial open complex. We also review the evidence that, at some but not all promoters, the initial open complex is stabilized and its lifetime greatly increased by a network of interactions involving the discriminator region of the nontemplate strand and mobile in-cleft and downstream elements of RNAP.

We review the strong formal parallels between the kinetics and mechanism of RNAP-promoter open complex formation and stabilization and the kinetics and mechanism of enzyme catalysis. Both mechanisms divide into three classes of steps with the central bottleneck step (catalysis, DNA opening; the focus of the mechanism) bracketed by reversible binding and conformational steps. In enzyme catalysis, reversible substrate binding and conformational steps are the focus of regulation by inhibitors, activators, and cooperative enzymes, while the central catalytic step is relatively unregulated. Evidence indicates that a similar, previously-unrecognized principle apply to regulation of transcription initiation. Most regulation of the rate of open complex formation occurs in the rapidly-reversible binding and closed-complex conformational steps that precede the central DNA opening step. Likewise, most regulation of open complex lifetime is in the steps that stabilize the initial open complex. The intrinsic DNA opening-closing step, the analog of the catalytic step, appears relatively universal and unregulated, with rates in both directions of approximately 1 s^−1^ at 37 °C.
